# Real-World Efficacy and Safety of Disitamab Vedotin (RC48-ADC) in the Treatment of HER2-Overexpressing Advanced Gastric/Gastroesophageal Junction Cancer

**DOI:** 10.3390/curroncol33010002

**Published:** 2025-12-19

**Authors:** Zhan Shi, Yan Wang, Yumeng Wang, Shutong Liu, Lianru Zhang, Kai Xin, Baorui Liu, Qin Liu

**Affiliations:** Department of Oncology, Nanjing Drum Tower Hospital, Affiliated Hospital of Medical School, Nanjing University, Nanjing 210008, China; zhanshi@sjtu.edu.cn (Z.S.);

**Keywords:** gastric/gastroesophageal junction cancer, HER2, disitamab vedotin, efficacy, safety

## Abstract

Objective: To evaluate the real-world efficacy and safety of disitamab vedotin (RC48-ADC) in patients with human epidermal growth factor receptor 2 (HER2) overexpression (immunohistochemistry [IHC] 2+ or 3+), advanced gastric/gastroesophageal junction cancer (GC/GEJC) with metastases who had received at least one line of prior systemic therapy. Patients and methods: Patients with HER2-overexpressing advanced or metastatic GC/GEJC who had previously received the anti-HER2 antibody-drug conjugate disitamab vedotin between December 2022 and April 2024 were enrolled in this study. The patients’ baseline characteristics, treatment procedures, and laboratory or imaging examinations were retrospectively collected and analyzed. The observation items included the objective response rate (ORR), disease control rate (DCR), progression-free survival (PFS), overall survival (OS), and treatment-related adverse events (TRAEs). Results: Of the 38 enrolled patients in the study, 27 were found to be HER2-positive. Most patients (29/38) received disitamab vedotin therapy as a third-line or subsequent treatment. A total of 68.4% of patients had previously received anti-HER2 therapy, and 13 patients underwent immunotherapy concurrently. The overall ORR and DCR were 31.6% and 65.8%, respectively. A higher ORR was observed in patients with a single metastatic site compared to those with multiple sites (53.3% vs. 17.4%, *p* = 0.022). In the general population, the median PFS was 6.5 months (95% confidence interval [CI] 3.3–9.8 months), and OS was 13.5 months (95% CI 9.0–17.9 months). The most common adverse event was anemia (89.5%), and eight patients suffered severe toxicities of grade ≥3. Conclusions: Disitamab vedotin exhibited encouraging anti-tumor effectiveness with a tolerable safety profile for advanced GC/GEJC patients with HER2 overexpression who had failed at least one line of systemic therapy in a real-world setting.

## Simple Summary

In this real-world study of patients with advanced stomach or gastroesophageal cancer that overproduces a protein called HER2, researchers tested a new targeted drug called disitamab vedotin after other treatments had failed. About one-third of these patients saw their tumors shrink and nearly two-thirds had their disease controlled. On average, patients lived for about 6.5 months before their cancer worsened and about 13.5 months overall after starting the treatment. The most common side effect was anemia, but the drug was generally well tolerated. These findings suggest that disitamab vedotin could be a promising new option for patients whose cancer continued progressing despite previous therapies, and they support further research and updates to treatment guidelines.

## 1. Introduction

Gastric cancer (GC) represents one of the most prevalent malignant cancers worldwide, ranking fifth in both incidence and mortality, as reported by GLOBOCAN 2022, with 358,700 new cases and 260,400 deaths in China [1,2]. Approximately 75% of GC patients are diagnosed at an advanced stage, for which chemotherapy-based systemic treatment has become the standard therapeutic approach. With the development of immunotherapy and targeted therapy, the treatment of GC has gradually evolved towards precision medicine. In addition, the discovery of molecular biomarkers, such as human epidermal growth factor receptor 2 (HER2), programmed cell death ligand 1 (PD-L1), microsatellite instability (MSI), Epstein–Barr virus (EBV), and tumor mutational burden (TMB), has facilitated the identification of the population likely to benefit from treatment, thereby improving the effectiveness of clinical interventions for patients with advanced GC [3].

HER2 is recognized as a crucial driver gene in gastric cancer and serves as the earliest and most mature biomarker for this disease. Patients with HER2-positive disease (immunohistochemistry [IHC] scores of 3+ or 2+ with fluorescence in situ hybridization [FISH]-positive) tend to have more aggressive disease and a poorer prognosis [4]. According to previous studies, the HER2-positive rate varies widely, ranging from 7.1% to 22.1% [5,6,7], and most IHC 2+ samples were negative by FISH testing (71.4–75.4%) [8,9]. In 2010, the ToGA study first reported that the combination of trastuzumab, an anti-HER2 monoclonal antibody, with chemotherapy resulted in an improvement in overall survival (OS) and progression-free survival (PFS) for patients with advanced HER2-positive GC or gastroesophageal junction cancer (GEJC) [10]. More recently, the phase III KEYNOTE-811 trial [11] revealed that the addition of immune checkpoint inhibitors (ICIs) to trastuzumab and chemotherapy significantly improved the survival benefits and treatment response rates in the first-line treatment of patients with locally advanced or metastatic HER2-positive GC/GEJC. Moreover, other anti-HER2 therapeutic agents, such as margetuximab [12], trastuzumab deruxtecan (DS8201) [13], ARX788 [14], ZW25 [15], and KN-026 [16], have also shown encouraging activity and manageable safety in patients with previously treated advanced HER2-positive GC/GEJC.

Disitamab vedotin (RC48), an innovative humanized HER2-targeted antibody-drug conjugate (ADC), has demonstrated promising efficacy and tolerable safety in HER2-overexpressing cancers (defined as IHC 2+/3+), including gastric cancer, breast cancer, and urothelial cancer [17]. It comprises a humanized anti-HER2 antibody (hertuzumab) conjugated to monomethyl auristatin E (MMAE) via a cleavable linker. Upon binding to HER2 receptors on the surface of tumor cells, the conjugate is rapidly internalized, and the linker is degraded in lysosomes, releasing free MMAE to induce mitotic arrest and apoptosis [17,18]. Notably, the hydrophobic payload can diffuse into neighboring cells and exert a bystander effect, which may be particularly relevant in HER2-heterogeneous or “HER2-low” tumors [19,20]. Preclinical evidence further indicates that HER2-targeted ADCs can elicit immunogenic cell death, enhance tumor antigen presentation, and increase expression of major histocompatibility complex (MHC) class I molecules and PD-L1, thereby potentiating anti-tumor immune responses [21,22,23]. A phase I study initially evaluated the toxicity and efficacy of disitamab vedotin in patients with HER2-positive solid tumors; within the gastric cancer subset of this trial, the objective response rate (ORR) and disease control rate (DCR) were 21.3% and 46.8%, respectively [24]. Furthermore, data from the C008 study [25] indicated an ORR of 24.8% and a DCR of 42.4%, with the median PFS and OS recorded at 4.1 months and 7.9 months, respectively, among HER2-overexpressing advanced GC/GEJC patients receiving third-line treatment or beyond. Subgroup analyses revealed that patients with HER2 IHC 2+, irrespective of their FISH status, exhibited a response consistent with that of the IHC 3+ cohort (ORR: 23.0% vs. 26.6%). In light of these findings, the National Medical Products Administration (NMPA) has approved disitamab vedotin to treat patients with locally advanced or metastatic gastric cancers characterized by HER2 overexpression who have undergone at least two lines of systemic chemotherapy. In addition, a multicenter, randomized, open-label, parallel-controlled phase III trial (C007, NCT04714190) is currently underway to further evaluate the anti-tumor activity and tolerability of disitamab vedotin compared to the standard treatment strategy determined by the physician.

However, clinical trials often establish stringent criteria for participant selection, potentially leading to the exclusion of individuals with specific characteristics that are frequently encountered in real-world populations, such as hepatic or renal impairment, poor performance status, ascites, and hydrothorax. To fill this gap, we conducted a descriptive retrospective analysis of patients diagnosed with HER2-overexpressing GC/GEJC who received treatment with disitamab vedotin at our institution. Additionally, we explored the association between the efficacy of disitamab vedotin and clinical factors.

## 2. Patients and Methods

### 2.1. Patients

We retrospectively collected the clinical data of patients who were administered disitamab vedotin at our institution between December 2022 and April 2024. Only patients who consented and signed the consent form were included. The inclusion criteria were as follows: advanced GC/GEJC with metastases; HER2 overexpression at initial diagnosis (IHC 3+ or 2+, regardless of FISH results); received at least two cycles of disitamab vedotin; underwent imaging assessments. Patients were excluded if they met the following criteria: received only one cycle of disitamab vedotin; did not undergo imaging examination; or lacked clinical data. The study was approved by the Ethics Committee of Nanjing Drum Tower Hospital (approval code: 2024-504-01; approval date: 22 August 2024). All procedures were performed following the ethical guidelines of the Declaration of Helsinki.

### 2.2. Treatment

All patients in the study were administered disitamab vedotin at a dosage of 120 mg intravenously for 60 min every 2 weeks. Additional concurrent therapeutic modalities, including immunotherapy, chemotherapy, targeted therapy, and/or radiotherapy, were determined at the physician’s discretion. The treatment with disitamab vedotin was maintained until disease progression, intolerable adverse events, or the patient’s death.

### 2.3. Efficacy and Safety Evaluation

During the treatment, all patients underwent imaging assessments via computed tomography (CT) and/or magnetic resonance imaging (MRI) at a designated interval of 6 weeks. In accordance with the Response Evaluation Criteria in Solid Tumors (RECIST) version 1.1, the treatment response was classified as complete response (CR), partial response (PR), stable disease (SD), and progressive disease (PD). The ORR was determined by aggregating the proportions of patients who were assessed as CR and PR, while the DCR was calculated as the ratio of patients achieving CR, PR, or SD. PFS was calculated from the start of disitamab vedotin therapy to the occurrence of disease progression or death, and OS was defined as the period from the start of disitamab vedotin therapy until either death or the end of follow-up. Treatment-related adverse events (TRAEs) were collected from the medical records and subsequently categorized according to the Common Terminology Criteria for Adverse Events (CTCAE) version 5.0.

### 2.4. Statistical Analysis

All statistical analyses were performed using SPSS version 22.0. Fisher’s exact test was applied to evaluate the differences in ORR and DCR across subgroups. PFS and OS were estimated using the Kaplan–Meier method, with comparisons between paired groups performed via the Log-rank test. *p* < 0.05 was considered statistically significant.

## 3. Results

### 3.1. Patients’ Characteristics

**Figure 1:**
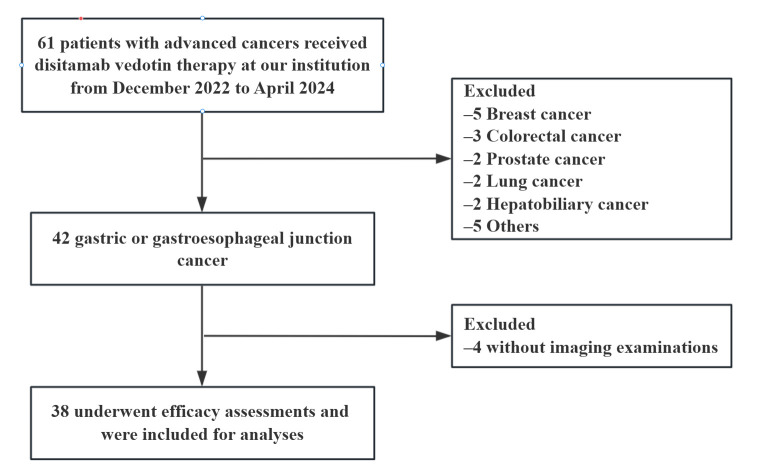
Flow diagram of patient selection.

As shown in Figure 1, from December 2022 to April 2024, 61 patients received disitamab vedotin treatment at our institution. Of these, 42 patients were diagnosed with GC/GEJC, and 38 patients underwent imaging assessments and were thus included in this descriptive retrospective study. The baseline characteristics of enrolled patients are presented in Table 1. In this study, the median age of participants was 66 years (range 34–84 years), with 27 males and 11 females. Among the 38 patients, 84.2% (32/38) were diagnosed with GC, and another 6 patients were diagnosed with GEJC. HER2 expression was positive in 27 patients, and 11 patients exhibited low HER2 expression (IHC 2+/FISH-negative). All patients had previously received at least one line of systemic treatment, including chemotherapy (38/38, 100%), immunotherapy (35/38, 92.1%), surgery (24/38, 63.2%), antiangiogenic therapy (6/38, 15.8%), and anti-HER2 therapy (26/38, 68.4%). Notably, a total of 29 patients underwent disitamab vedotin treatment as third-line or later treatment. Most patients (60.5%) exhibited multiple metastatic sites, with the lymph nodes (71.1%) representing the most prevalent site of metastasis.

### 3.2. Efficacy

**Figure 2:**
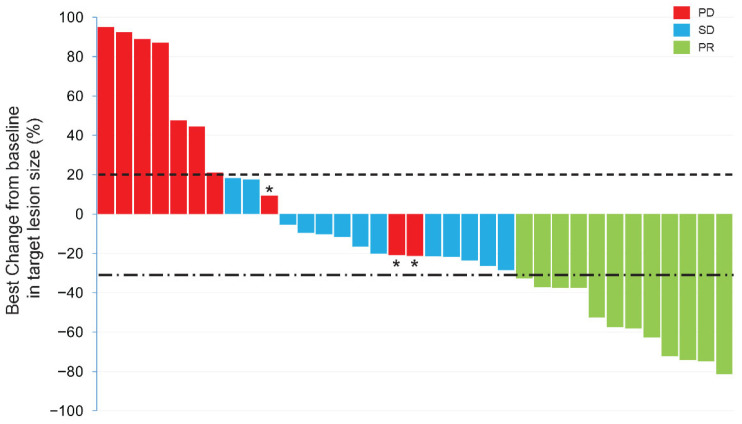
The best response change from baseline in the sum of the diameters of target lesions. The dotted horizontal line indicates the +20% threshold (PD), and the dash-dotted horizontal line indicates the −30% threshold (PR). * means patients had at least one new lesion during the treatment. Abbreviations: PR, partial response; SD, stable disease; PD, progressive disease.

Overall, 35 of the 38 patients presented at least one measurable lesion at baseline, while an additional 3 patients had peritoneal or bone metastases. According to the RECIST version 1.1, 12 patients were evaluated as PR, 13 had SD, and 13 exhibited PD, including 6 patients with the emergence of one or more new lesions. The overall ORR and DCR were 31.6% and 65.8%, respectively (Table 2, Figure 2). In addition, we analyzed the relationship between the treatment efficacy and various baseline characteristics, such as the primary tumor location, the number of metastatic sites, prior line of treatment, HER2 status, prior anti-HER2 therapy, and concurrent treatment regimens. Subgroup analyses indicated that patients with one metastatic site achieved a higher ORR (53.3% vs. 17.4%, *p* = 0.022), while the DCR was comparable between groups (73.3% vs. 60.9%, *p* = 0.435), and no notable difference in treatment response was observed between other paired groups. Interestingly, a higher DCR was observed in patients diagnosed with GEJC compared to those with GC (100% vs. 59.3%, *p* = 0.054), although the underlying mechanism for this observation remains unclear.

**Figure 3:**
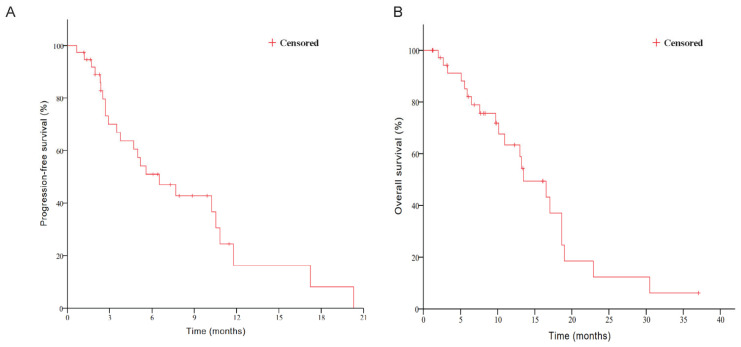

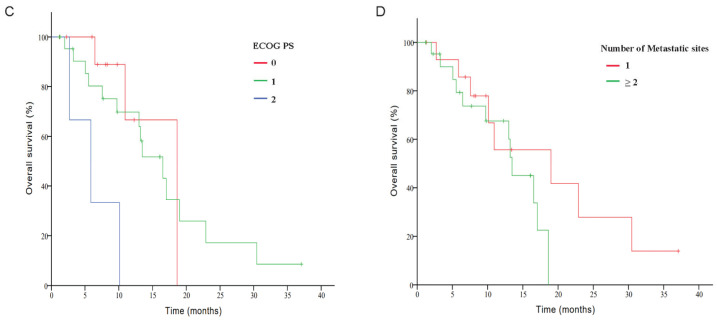
Kaplan–Meier curves for PFS (**A**,**B**) and OS stratified by ECOG PS (**C**) and number of metastatic sites (**D**). Abbreviations: PFS, progression-free survival; OS, overall survival; ECOG PS, Eastern Cooperative Oncology Group performance status.

Towards the end of the follow-up period on 6 August 2024, the median PFS for the enrolled cohort was 6.5 months (95% confidence interval [CI] 3.3–9.8 months), and the median OS was 13.5 months (95% CI, 9.0–17.9 months) (Figure 3A,B). Our analysis revealed that patients with an Eastern Cooperative Oncology Group (ECOG) performance status (PS) of 0 experienced prolonged survival compared to those with an ECOG score of 1 or 2, exhibiting median OS durations of 18.6, 16.5, and 5.9 months, respectively (*p* = 0.009) (Figure 3C). Furthermore, patients with only a single metastatic site showed a trend toward improved overall survival in comparison to those with multiple metastatic sites (19.0 vs. 13.5 months), while this difference did not reach statistical significance (*p* = 0.119) (Figure 3D). No other subgroups displayed significant advantages in terms of either PFS or OS.

### 3.3. Safety

The TRAEs are listed in Table 3. Overall, all patients experienced at least one TRAE, with the majority of TRAEs graded 1 or 2. Eight patients encountered severe TRAEs (≥grade 3). During the treatment, the most common hematologic events were anemia (34/38, 89.5%), lymphocytopenia (31/38, 81.6%), and hypoalbuminemia (27/38, 71.1%). Leukopenia and neutropenia were observed in 20 and 16 patients, respectively. Decreased appetite occurred in 24 patients, with three patients suffering from grade 3 or higher. Furthermore, 7 patients developed rashes, with one case being particularly severe, resulting in the discontinuation of disitamab vedotin. No treatment-related deaths occurred during the study.

## 4. Discussion

The present study evaluated the efficacy and safety of disitamab vedotin as second-line therapy and beyond in advanced GC/GEJC patients with metastases characterized by HER2 overexpression. In this study, we observed an encouraging tumor response with an ORR of 31.6% and a DCR of 65.8%, which further validated the promising efficacy of disitamab vedotin for patients with HER2-overexpressing advanced GC/GEJC in a real-world setting. Previous studies have demonstrated the synergistic effects of different antitumor treatment modalities [26,27,28]. Over 50% of participants in this study received concurrent multimodal treatment, such as immunotherapy, chemotherapy, radiotherapy, and antiangiogenic therapy, which may result in a higher treatment response rate compared to the phase II C008 study, which reported an ORR of 24.8% and a DCR of 42.4%. Additionally, patients undergoing combination therapy achieved prolonged overall survival benefits compared to those receiving disitamab vedotin alone (16.5 vs. 13.5 months, *p* = 0.291), whereas no statistically significant differences were identified between the aforementioned treatment modalities in this study, indicating that further research is needed to determine the optimal concurrent treatment regimen.

In 2023, the phase II DESTINY-Gastric02 trial provided novel evidence that trastuzumab deruxtecan (a HER2-targeted ADC) was an effective and safe second-line therapy for HER2-positive advanced GC/GEJC patients. This trial reported an ORR of 41.8%, alongside a PFS of 5.6 months and OS of 12.1 months [29]. Additionally, a propensity score-matched comparative study demonstrated that trastuzumab deruxtecan improved overall survival when compared to the combination of ramucirumab and paclitaxel as a second-line therapy in patients with HER2-positive GC/GEJC, yielding a median OS of 11.6 versus 6.2 months (hazard ratio [HR] = 0.39, *p* < 0.0001) [30]. During this study period, no standardized second-line HER2-targeted therapy had been established for patients with HER2-positive GC/GEJC who had experienced disease progression following first-line trastuzumab-based treatment. The decision regarding second-line therapy was made individually by physicians, based on a comprehensive evaluation of each patient’s disease status, prior treatment response, and the availability of alternative therapeutic options. In our investigation, nine patients received disitamab vedotin as second-line therapy, revealing a favorable tumor response characterized by an ORR of 44.4% and a DCR of 88.9%. Analyses of survival benefits showed that the median OS for these patients was 17.0 months (95% CI, 10.1–24.0 months), with a median PFS of 7.7 months (95% CI, 5.3–10.1 months) (Supplementary Figure S1). To our knowledge, this is the first report to evaluate the efficacy of disitamab vedotin as a second-line therapy in advanced GC/GEJC patients with HER2 overexpression. The treatment response and survival outcomes observed in the present study were non-inferior to previous reports, suggesting the potential for disitamab vedotin to be applied in earlier lines of therapy in the future.

As shown in Table 1, 26 of the enrolled patients had previously received anti-HER2 therapy, which consisted of trastuzumab (23/26), pyrotinib (2/26), and ARX-788 (1/26), and no significant differences were observed between patients who had received prior anti-HER2 therapy or not (ORR: 33.3% vs. 25.0%, *p* = 0.553; DCR: 65.4% vs. 66.7%, *p* = 0.938), which is consistent with the results of previous reports. This result suggests that prior anti-HER2 therapy does not significantly affect the efficacy of disitamab vedotin treatment in HER2-overexpressing GC/GEJC patients and that disitamab vedotin may be able to reverse the trastuzumab resistance. Additionally, we found that patients who had not undergone anti-HER2 therapy achieved superior survival benefits (median PFS: 10.8 months, 95% CI, 2.0–19.7 months; median OS: 19.0 months, 95% CI, 10.1–27.9 months) compared to those who had received anti-HER2 therapy previously (median PFS: 6.5 months, 95% CI, 2.0–10.1 months; median OS: 13.5 months, 95% CI, 8.8–18.2 months), but the difference was not statistically significant (PFS: *p* = 0.571; OS: *p* = 0.178) (Supplementary Figure S2). The underlying mechanism may be associated with the dynamic alteration of HER2 status throughout disease progression. Prior research has indicated that a reduction or complete loss of HER2 expression occurs in 30% to 60% of patients with advanced or recurrent GC who have developed resistance to trastuzumab [31]. This decline in HER2 expression is likely driven by tumor heterogeneity and selective pressure exerted by prior therapy, potentially leading to reduced effectiveness of subsequent HER2-targeted treatments [32].

Preclinical evidence has indicated that HER2-ADC therapy stimulates the production of interferon-gamma (IFN-γ)-producing CD8+ antitumor T cells, leading to an increased expression of MHC class I and PD-L1 [33,34,35]. Furthermore, ADCs have the potential to exhibit bystander activity, thereby overcoming the challenges posed by the heterogeneous nature of the tumor microenvironment [36]. The RC48-C014 study demonstrated that disitamab vedotin plus programmed cell death-1 (PD-1) inhibitor yielded better PFS and ORR compared to disitamab vedotin alone in locally advanced or metastatic urothelial carcinoma patients [37]. In 2023, Nie and colleagues reported that the combination of disitamab vedotin and ICIs exhibited superior clinical advantages than disitamab vedotin monotherapy in both HER2-positive and HER2-low (IHC 1+/IHC 2+ and FISH-negative) gastric cancer [38]. Recently, a multicenter phase I trial found that disitamab vedotin combined with the anti-PD-1 antibody showed promising efficacy and manageable safety for patients with HER2-expressing solid tumors, including GC/GEJC [39]. Additionally, the RCTS trial [40] highlighted the remarkable efficacy of disitamab vedotin plus PD-1 inhibitors and chemotherapy as the first-line therapy for HER2-overexpressing advanced GC/GEJC patients. As shown in the Results, 13 patients received immunotherapy as the concurrent treatment modality in this study, but we did not observe the synergistic effects when comparing the combination of disitamab vedotin and immunotherapy to other treatment regimens, as demonstrated by the ORR of 30.8% versus 32.0% (*p* = 0.938) and DCR of 61.5% versus 68.0% (*p* = 0.690). One potential reason for this phenomenon could be the high prevalence of prior ICI therapy among the study population. It is noteworthy that 92.1% (35 out of 38) of the enrolled patients had undergone immunotherapy before this study, and there is currently a lack of consensus regarding the effectiveness of ICI rechallenge for patients who have tolerated initial immunotherapy [41].

To optimize cost-effectiveness and minimize drug wastage, in our clinical practice, we typically administer two vials of disitamab vedotin every two weeks at a fixed dose of 120 mg instead of adhering to the recommended dosage of 2.5 mg/kg. The overall safety profile of disitamab vedotin in this condition was manageable, as no unforeseen adverse events occurred throughout the treatment period. Notably, 57.9% (22 out of 38) of the patients received concomitant therapies in this study, and only one patient discontinued treatment due to the occurrence of a severe rash. In alignment with previous reports, the prevalent adverse events associated with disitamab vedotin in this real-world setting were bone marrow suppression and decreased appetite, while the occurrence of increased alanine aminotransferase (ALT) or aspartate aminotransferase (AST) was observed less frequently.

There are several limitations in the current study. First, the heterogeneity of patients’ baseline characteristics and treatment procedures may lead to potential bias in the findings. Second, the relatively small sample size, combined with a single-center and retrospective study design, limits the external validity and generalizability of the findings. Third, HER2 status in metastatic lesions at the time of disitamab vedotin administration was not systematically reassessed, and any dynamic changes in HER2 expression during disease progression could not be captured, which may potentially affect the accuracy of efficacy interpretation. Finally, due to the incomplete medical records inherent in the retrospective design, it is difficult to capture all the non-hematological side effects.

In conclusion, our study validated the efficacy and safety of disitamab vedotin in advanced GC/GEJC patients exhibiting HER2 overexpression, who had previously received at least one failed systemic treatment under real-world conditions. The results indicate that disitamab vedotin provides promising anti-tumor activity and manageable tolerance, irrespective of prior treatments or lines of therapy.

## Figures and Tables

**Table 1:** Patients’ characteristics.

Characteristics	No. of Patients (N = 38)	%
Age (years)		
Median	66 (range 34–84 years)	
<65	18	47.4
≥65	20	52.6
Sex		
Male	27	71.1
Female	11	28.9
ECOG PS		
0	11	28.9
1	24	63.2
2	3	7.9
Family history of cancer		
Yes	6	15.8
No	32	84.2
Primary tumor site		
Gastric	32	84.2
Gastroesophageal junction	6	15.8
HER2 status		
IHC 2+ and FISH-negative	11	28.9
IHC 2+ and FISH-positive	3	7.9
IHC 3+	24	63.2
Prior anti-HER2 therapy		
Yes	26	68.4
No	12	31.6
Prior surgery		
Yes	24	63.2
No	14	36.8
Prior chemotherapy		
Yes	38	100
No	0	0
Prior radiotherapy		
Yes	12	31.6
No	26	68.4
Prior immunotherapy		
Yes	35	92.1
No	3	7.9
Prior antiangiogenic therapy		
Yes	6	15.8
No	32	84.2
Number of treatment lines		
2	9	23.7
≥3	29	76.3
Concurrent therapy		
Chemotherapy	4	10.5
Radiotherapy	5	13.2
Immunotherapy	13	34.2
Targeted therapy	6	15.8
None	16	42.1
Number of metastatic sites		
1	15	39.5
≥2	23	60.5
Metastatic sites		
Liver	15	39.5
Lung	7	18.4
Bone	6	15.8
Lymph nodes	27	71.1
Adrenal gland	2	5.3
Others	11	28.9

Abbreviations: ECOG PS, Eastern Cooperative Oncology Group performance status; HER2, human epidermal growth factor receptor 2; IHC, immunohistochemistry; FISH, fluorescence in situ hybridization.

**Table 2:** Treatment response.

Parameters	Best Tumor Response				ORR	*p*	DCR	*p*
	CR	PR	SD	PD				
Total	0	12	13	13	31.6		65.8	
Primary tumor site						0.920		0.054
GC	0	10	9	13	31.3		59.4	
GEJC	0	2	4	0	33.3		100	
Number of metastatic sites						0.022		0.435
1	0	8	3	4	53.3		73.3	
≥2	0	4	10	9	17.4		60.9	
HER2 status						0.715		0.858
Positive	0	9	9	9	33.3		66.7	
Negative	0	3	4	4	27.3		63.6	
Treatment lines						0.342		0.095
2	0	4	4	1	44.4		88.9	
≥3	0	8	9	12	27.6		58.6	
Prior anti-HER2 therapy						0.553		0.938
Yes	0	9	8	9	34.6		65.4	
No	0	3	5	4	25.0		66.7	
Concurrent ICIs therapy						0.938		0.690
Yes	0	4	4	5	30.8		61.5	
No	0	8	9	8	32.0		68.0	

Abbreviations: CR, complete response; PR, partial response; SD, stable disease; PD, progressive disease; ORR, overall response rate; DCR, disease control rate; GC, gastric cancer; GEJC, gastroesophageal junction cancer; HER2, human epidermal growth factor receptor 2; ICIs, immune checkpoint inhibitors.

**Table 3:** Treatment-related adverse events.

TRAEs	Grade (N = 38)			
	Any Grades (%)	1 (%)	2 (%)	≥3 (%)
Hematologic				
Anemia	34 (89.5)	22 (57.9)	7 (18.4)	5 (13.2)
Lymphocytopenia	31 (81.6)	11 (28.9)	13 (34.2)	7 (18.4)
Hypoalbuminemia	27 (71.1)	25 (65.8)	2 (5.3)	0
Increased LDH	22 (57.9)	15 (39.5)	7 (18.4)	0
Leukopenia	20 (52.6)	4 (10.5)	15 (39.5)	1 (2.6)
Increased γGT	19 (50.0)	6 (15.8)	7 (18.4)	6 (15.8)
Neutropenia	16 (42.1)	3 (7.9)	9 (23.7)	4 (10.5)
Hypophosphatemia	16 (42.1)	10 (26.3)	5 (13.2)	1 (2.6)
Hypothyroidism	13 (34.2)	12 (31.6)	1 (2.6)	0
Hyperglycemia	11 (28.9)	7 (18.4)	2 (5.3)	2 (5.3)
Hypokalemia	10 (26.3)	3 (7.9)	5 (13.2)	2 (5.3)
Hyponatremia	10 (26.3)	7 (18.4)	2 (5.3)	1 (2.6)
Thrombocytopenia	8 (21.1)	5 (13.2)	0	3 (7.9)
Increased ALT	7 (18.4)	6 (15.8)	1 (2.6)	0
Hyperlipemia	7 (18.4)	6 (15.8)	0	1 (2.6)
Hypercholesteremia	7 (18.4)	7 (18.4)	0	0
Increased AST	6 (15.8)	4 (10.5)	1 (2.6)	1 (2.6)
Hyperbilirubinemia	5 (13.2)	2 (5.3)	2 (5.3)	1 (2.6)
Increased ALP	2 (5.3)	2 (5.3)	0	0
Non-hematologic				
Decreased appetite	24 (63.2)	17 (44.7)	4 (10.5)	3 (7.9)
Fatigue	11 (28.9)	8 (21.1)	3 (7.9)	0
Vomiting	10 (26.3)	7 (18.4)	2 (5.3)	1 (2.6)
Constipation	9 (23.7)	7 (18.4)	2 (5.3)	0
Abdominal pain	9 (23.7)	4 (10.5)	3 (7.9)	2 (5.3)
Neuropathy peripheral	8 (21.1)	5 (13.2)	3 (7.9)	0
Pruritus	7 (18.4)	3 (7.9)	3 (7.9)	1 (2.6)
Nausea	7 (18.4)	4 (10.5)	1 (2.6)	2 (5.3)
Pyrexia	7 (18.4)	6 (15.8)	1 (2.6)	0
Rash	7 (18.4)	5 (13.2)	1 (2.6)	1 (2.6)
Diarrhea	6 (15.8)	5 (13.2)	0	1 (2.6)
Muscle pain/joint pain	4 (10.5)	1 (2.6)	2 (5.3)	1 (2.6)
Weight loss	2 (5.3)	1 (2.6)	1 (2.6)	0

Abbreviations: TRAEs, treatment-related adverse events; LDH, lactate dehydrogenase; γGT, gamma-glutamyltransferase; ALT, alanine aminotransferase; AST, aspartate aminotransferase; ALP, alkaline phosphatase.

## Data Availability

The dataset used and/or analyzed during current study are available from the corresponding author on reasonable request.

## Supplementary Materials

The following supporting information can be downloaded at: https://www.mdpi.com/article/10.3390/curroncol33010002/s1, Supplementary Figure S1. Kaplan–Meier curves for PFS (A) and OS (B) according to the number of treatment lines. Abbreviations: PFS, progression-free survival; OS, overall survival. Supplementary Figure S2. Kaplan–Meier curves for PFS (A) and OS (B) according to prior anti-HER2 therapy. Abbreviations: PFS, progression-free survival; OS, overall survival; HER2, human epidermal growth factor receptor 2.

## References

[1] Bray F., Laversanne M., Sung H., Ferlay J., Siegel R.L., Soerjomataram I., Jemal A. (2024). Global cancer statistics 2022: GLOBOCAN estimates of incidence and mortality worldwide for 36 cancers in 185 countries. CA A Cancer J. Clin..

[2] Han B., Zheng R., Zeng H., Wang S., Sun K., Chen R., Li L., Wei W., He J. (2024). Cancer incidence and mortality in China, 2022. J. Natl. Cancer Cent..

[3] Zhang Z., Huang J., Li Y., Yan H., Xie J., Wang J., Zhao B. (2024). Global burden, risk factors, clinicopathological characteristics, molecular biomarkers and outcomes of microsatellite instability-high gastric cancer. Aging.

[4] Smyth E.C., Nilsson M., Grabsch H.I., van Grieken N.C., Lordick F. (2020). Gastric cancer. Lancet.

[5] Yang J., Shi Z., Zhang X., Liu Q., Cui X., Li L., Liu B., Wei J. (2023). Real-world clinical outcomes of the combination of anti-PD-1 antibody, trastuzumab, and chemotherapy for HER2-positive gastric/gastroesophageal junction cancer. Cancer Med..

[6] Takehana T., Kunitomo K., Kono K., Kitahara F., Iizuka H., Matsumoto Y., Fujino M.A., Ooi A. (2002). Status of c-erbB-2 in gastric adenocarcinoma: A comparative study of immunohistochemistry, fluorescence in situ hybridization and enzyme-linked immuno-sorbent assay. Int. J. Cancer.

[7] Van Cutsem E., Bang Y.J., Feng-Yi F., Xu J.M., Lee K.W., Jiao S.C., Chong J.L., López-Sanchez R.I., Price T., Gladkov O. (2015). HER2 screening data from ToGA: Targeting HER2 in gastric and gastroesophageal junction cancer. Gastric Cancer.

[8] Huang D., Li Z.S., Fan X.S., Wu H.M., Liu J.P., Sun W.Y., Li S.S., Hou Y.Y., Nie X., Li J. (2018). HER2 status in gastric adenocarcinoma of Chinese: A multicenter study of 40 842 patients. Zhonghua Bing Li Xue Za Zhi.

[9] Kim W.-H., Gomez-Izquierdo L., Vilardell F., Chu K.-M., Soucy G., dos Santos L.V., Monges G., Viale G., Brito M.J., Osborne S. (2018). HER2 Status in Gastric and Gastroesophageal Junction Cancer: Results of the Large, Multinational HER-EAGLE Study. Appl. Immunohistochem. Mol. Morphol..

[10] Bang Y.-J., Van Cutsem E., Feyereislova A., Chung H.C., Shen L., Sawaki A., Lordick F., Ohtsu A., Omuro Y., Satoh T. (2010). Trastuzumab in combination with chemotherapy versus chemotherapy alone for treatment of HER2-positive advanced gastric or gastro-oesophageal junction cancer (ToGA): A phase 3, open-label, randomised controlled trial. Lancet.

[11] Janjigian Y.Y., Kawazoe A., Bai Y., Xu J., Lonardi S., Metges J.P., Yanez P., Wyrwicz L.S., Shen L., Ostapenko Y. (2023). Pembrolizumab plus trastuzumab and chemotherapy for HER2-positive gastric or gastro-oesophageal junction adenocarcinoma: Interim analyses from the phase 3 KEYNOTE-811 randomised placebo-controlled trial. Lancet.

[12] Catenacci D.V.T., Kang Y.-K., Park H., Uronis H.E., Lee K.-W., Ng M.C.H., Enzinger P.C., Park S.H., Gold P.J., Lacy J. (2020). Margetuximab plus pembrolizumab in patients with previously treated, HER2-positive gastro-oesophageal adenocarcinoma (CP-MGAH22–05): A single-arm, phase 1b–2 trial. Lancet Oncol..

[13] Shitara K., Bang Y.-J., Iwasa S., Sugimoto N., Ryu M.-H., Sakai D., Chung H.-C., Kawakami H., Yabusaki H., Lee J. (2020). Trastuzumab Deruxtecan in Previously Treated HER2-Positive Gastric Cancer. N. Engl. J. Med..

[14] Zhang Y., Qiu M.-Z., Wang J.-F., Zhang Y.-Q., Shen A., Yuan X.-L., Zhang T., Wei X.-L., Zhao H.-Y., Wang D.-S. (2022). Phase 1 multicenter, dose-expansion study of ARX788 as monotherapy in HER2-positive advanced gastric and gastroesophageal junction adenocarcinoma. Cell Rep. Med..

[15] Meric-Bernstam F., Beeram M., Mayordomo J.I., Hanna D.L., Ajani J.A., Murphy M.A.B., Murthy R.K., Piha-Paul S.A., Bauer T.M., Bendell J.C. (2018). Single agent activity of ZW25, a HER2-targeted bispecific antibody, in heavily pretreated HER2-expressing cancers. J. Clin. Oncol..

[16] Xu J., Ying J., Liu R., Wu J., Ye F., Xu N., Zhang Y., Zhao R., Xiang X., Wang J. (2022). KN026 (anti-HER2 bispecific antibody) in patients with previously treated, advanced HER2-expressing gastric or gastroesophageal junction cancer. Eur. J. Cancer.

[17] Shi F., Liu Y., Zhou X., Shen P., Xue R., Zhang M. (2022). Disitamab vedotin: A novel antibody-drug conjugates for cancer therapy. Drug Deliv..

[18] Mark C., Lee J.S., Cui X., Yuan Y. (2023). Antibody-Drug Conjugates in Breast Cancer: Current Status and Future Directions. Int. J. Mol. Sci..

[19] Giugliano F., Corti C., Tarantino P., Michelini F., Curigliano G. (2022). Bystander effect of antibody-drug conjugates: Fact or fiction. Curr. Oncol. Rep..

[20] Martín M., Pandiella A., Vargas-Castrillón E., Díaz-Rodríguez E., Iglesias-Hernangómez T., Martínez Cano C., Fernández-Cuesta I., Winkow E., Perelló M.F. (2024). Trastuzumab deruxtecan in breast cancer. Crit. Rev. Oncol./Hematol.

[21] Tsao L.C., Wang J.S., Ma X., Sodhi S., Ragusa J.V., Liu B., McBane J., Wang T., Wei J., Liu C.X. (2025). Effective extracellular payload release and immunomodulatory interactions govern the therapeutic effect of trastuzumab deruxtecan (T-DXd). Nat. Commun..

[22] Nakajima S., Mimura K., Matsumoto T., Thar Min A.K., Ito M., Nakano H., Neupane P., Kanke Y., Okayama H., Saito M. (2021). The effects of T-DXd on the expression of HLA class I and chemokines CXCL9/10/11 in HER2-overexpressing gastric cancer cells. Sci. Rep..

[23] Wu X., Xu L., Li X., Zhou Y., Han X., Zhang W., Wang W., Guo W., Liu W., Xu Q. (2023). A HER2-targeting antibody-MMAE conjugate RC48 sensitizes immunotherapy in HER2-positive colon cancer by triggering the cGAS-STING pathway. Cell Death Dis..

[24] Xu Y., Wang Y., Gong J., Zhang X., Peng Z., Sheng X., Mao C., Fan Q., Bai Y., Ba Y. (2021). Phase I study of the recombinant humanized anti-HER2 monoclonal antibody–MMAE conjugate RC48-ADC in patients with HER2-positive advanced solid tumors. Gastric Cancer.

[25] Peng Z., Liu T., Wei J., Wang A., He Y., Yang L., Zhang X., Fan N., Luo S., Li Z. (2021). Efficacy and safety of a novel anti-HER2 therapeutic antibody RC48 in patients with HER2-overexpressing, locally advanced or metastatic gastric or gastroesophageal junction cancer: A single-arm phase II study. Cancer Commun..

[26] Cui J., He Y., Zhu F., Gong W., Zuo R., Wang Y., Luo Y., Chen L., Wang C., Huo G. (2023). Inetetamab, a novel anti-HER2 monoclonal antibody, exhibits potent synergistic anticancer effects with cisplatin by inducing pyroptosis in lung adenocarcinoma. Int. J. Biol. Sci..

[27] Fu R., Qi R., Xiong H., Lei X., Jiang Y., He J., Chen F., Zhang L., Qiu D., Chen Y. (2024). Combination therapy with oncolytic virus and T cells or mRNA vaccine amplifies antitumor effects. Signal Transduct. Target. Ther..

[28] Ito Y., Yamada D., Kobayashi S., Sasaki K., Iwagami Y., Tomimaru Y., Asaoka T., Noda T., Takahashi H., Shimizu J. (2024). The combination of gemcitabine plus an anti-FGFR inhibitor can have a synergistic antitumor effect on FGF-activating cholangiocarcinoma. Cancer Lett..

[29] Van Cutsem E., di Bartolomeo M., Smyth E., Chau I., Park H., Siena S., Lonardi S., Wainberg Z.A., Ajani J., Chao J. (2023). Trastuzumab deruxtecan in patients in the USA and Europe with HER2-positive advanced gastric or gastroesophageal junction cancer with disease progression on or after a trastuzumab-containing regimen (DESTINY-Gastric02): Primary and updated analyses from a single-arm, phase 2 study. Lancet Oncol..

[30] Verhoeven R.H.A., Kuijper S.C., Lordick F., Slingerland M., Qin A., van Laarhoven H.W.M. (2023). 1574P Trastuzumab deruxtecan versus ramucirumab and paclitaxel as second-line therapy for patients with her2-positive gastric or gastro-esophageal junction adenocarcinoma: A propensity score matched comparison. Ann. Oncol..

[31] Scheck M.K., Hofheinz R.D., Lorenzen S. (2024). HER2-Positive Gastric Cancer and Antibody Treatment: State of the Art and Future Developments. Cancers.

[32] Ratti M., Citterio C., Orlandi E., Vecchia S., Anselmi E., Toscani I., Rotolo M., Salati M., Ghidini M. (2025). Fighting HER2 in Gastric Cancer: Current Approaches and Future Landscapes. Int. J. Mol. Sci..

[33] Stagg J., Loi S., Divisekera U., Ngiow S.F., Duret H., Yagita H., Teng M.W., Smyth M.J. (2011). Anti–ErbB-2 mAb therapy requires type I and II interferons and synergizes with anti–PD-1 or anti-CD137 mAb therapy. Proc. Natl. Acad. Sci. USA.

[34] Bianchini G., Gianni L. (2014). The immune system and response to HER2-targeted treatment in breast cancer. Lancet Oncol..

[35] Huang L., Wang R., Xie K., Zhang J., Tao F., Pi C., Feng Y., Gu H., Fang J. (2021). A HER2 target antibody drug conjugate combined with anti-PD-(L)1 treatment eliminates hHER2+ tumors in hPD-1 transgenic mouse model and contributes immune memory formation. Breast Cancer Res. Treat..

[36] Wei Q., Yang T., Zhu J., Zhang Z., Yang L., Zhang Y., Hu C., Chen J., Wang J., Tian X. (2024). Spatiotemporal Quantification of HER2-targeting Antibody-Drug Conjugate Bystander Activity and Enhancement of Solid Tumor Penetration. Clin. Cancer Res. Off. J. Am. Assoc. Cancer Res..

[37] Sheng X., Zhou L., He Z., Guo H., Yan X., Li S., Xu H., Li J., Chi Z., Si L. (2022). Preliminary results of a phase Ib/II combination study of RC48-ADC, a novel humanized anti-HER2 antibody-drug conjugate (ADC) with toripalimab, a humanized IgG4 mAb against programmed death-1 (PD-1) in patients with locally advanced or metastatic urothelial carcinoma. J. Clin. Oncol..

[38] Nie C., Xu W., Guo Y., Gao X., Lv H., Chen B., Wang J., Liu Y., Zhao J., Wang S. (2023). Immune checkpoint inhibitors enhanced the antitumor efficacy of disitamab vedotin for patients with HER2-positive or HER2-low advanced or metastatic gastric cancer: A multicenter real-world study. BMC Cancer.

[39] Wang Y., Gong J., Wang A., Wei J., Peng Z., Wang X., Zhou J., Qi C., Liu D., Li J. (2024). Disitamab vedotin (RC48) plus toripalimab for HER2-expressing advanced gastric or gastroesophageal junction and other solid tumours: A multicentre, open label, dose escalation and expansion phase 1 trial. Eclinicalmedicine.

[40] Li S., Liu Z., Liu Y., Li K., Cong L., Cao F., Liu A., Liu H., Li L., Qu L. (2024). Efficacy of disitamab vedotin (RC48) plus tislelizumab and S-1 as first-line therapy for HER2-overexpressing advanced stomach or gastroesophageal junction adenocarcinoma: A multicenter, single-arm, phase II trial (RCTS). J. Clin. Oncol..

[41] Hu H., Wang K., Jia R., Zeng Z.-X., Zhu M., Deng Y.-L., Xiong Z.-J., Tang J.-N., Xie H., Wang Y. (2023). Current Status in Rechallenge of Immunotherapy. Int. J. Biol. Sci..

