# Supplementary figures and images for: Real-World Efficacy and Safety of Disitamab Vedotin (RC48-ADC) in the Treatment of HER2-Overexpressing Advanced Gastric/Gastroesophageal Junction Cancer
Source: Curr Oncol. 2025 Dec 19;33(1):2. doi: 10.3390/curroncol33010002 (PMC12839656; doi:10.3390/curroncol33010002)

Supplementary Figure 1

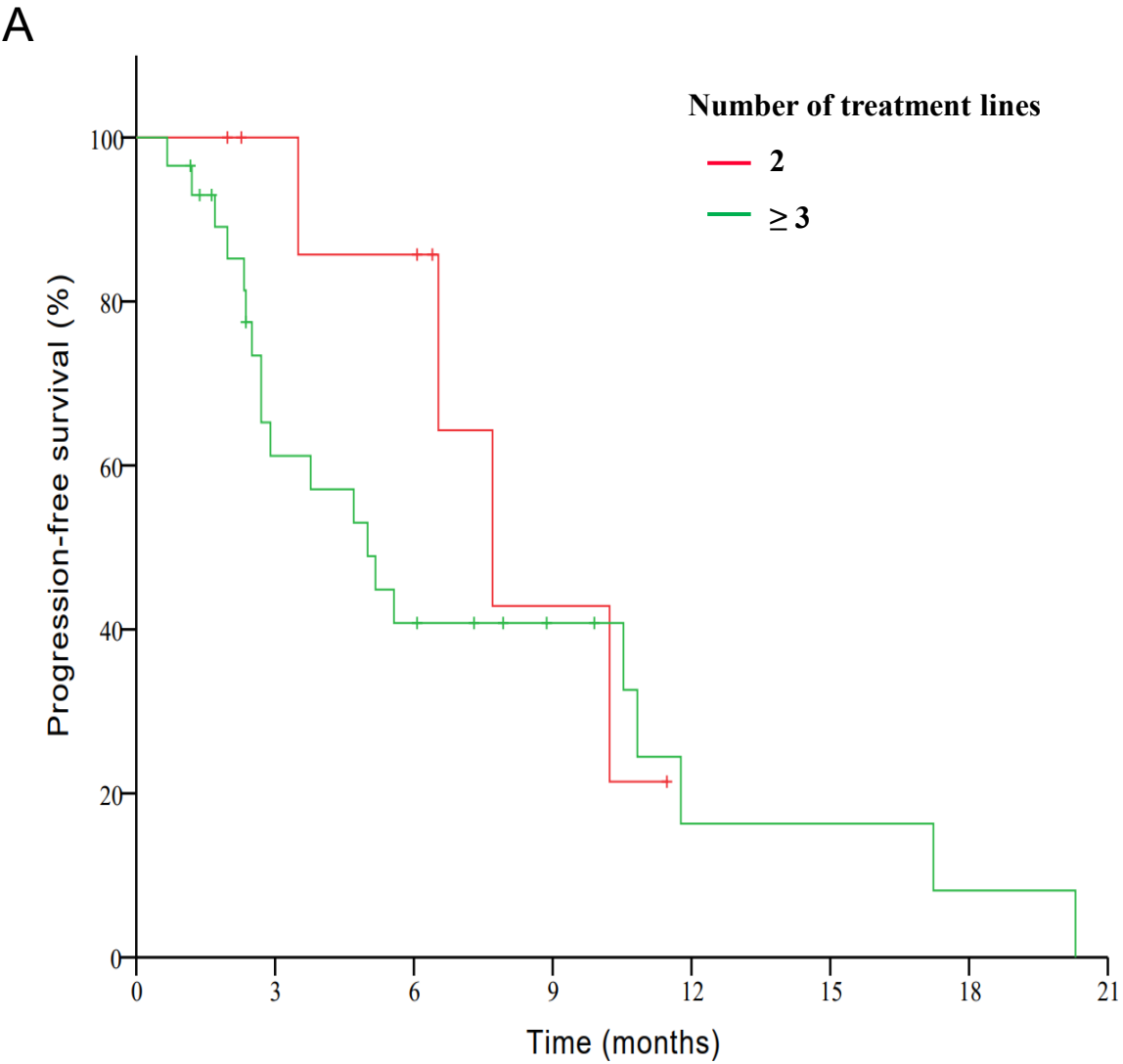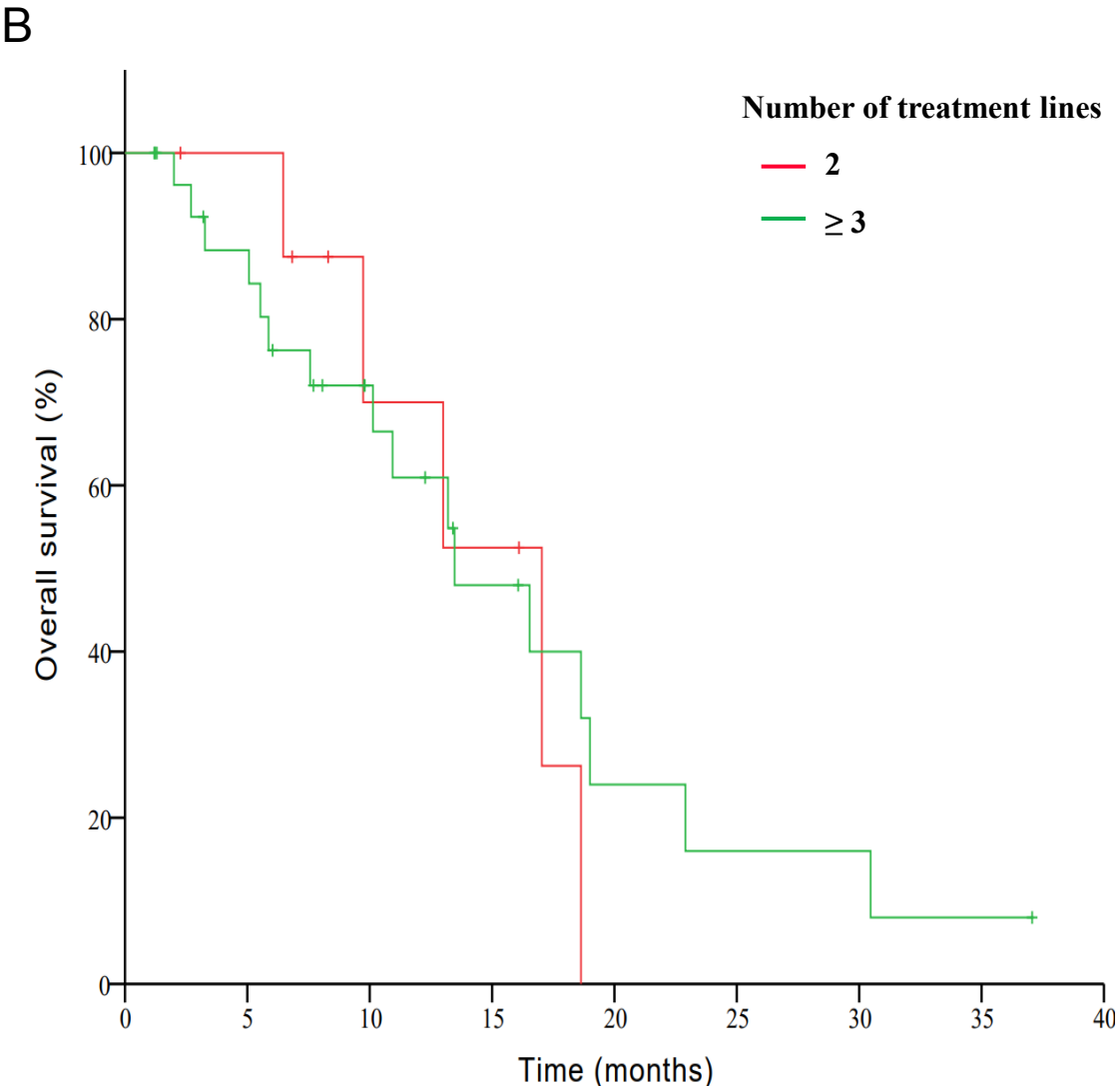

Supplementary Figure 2

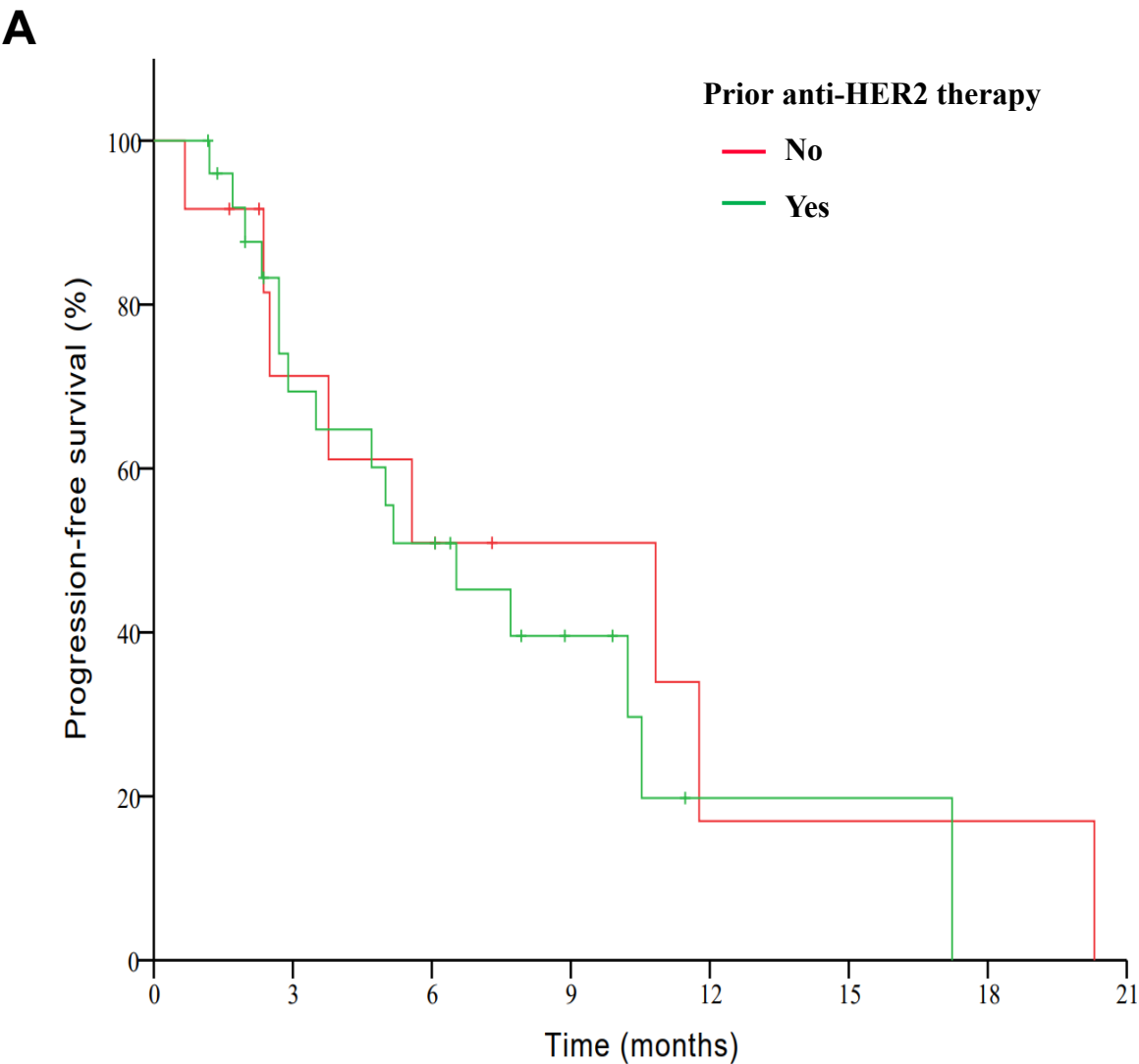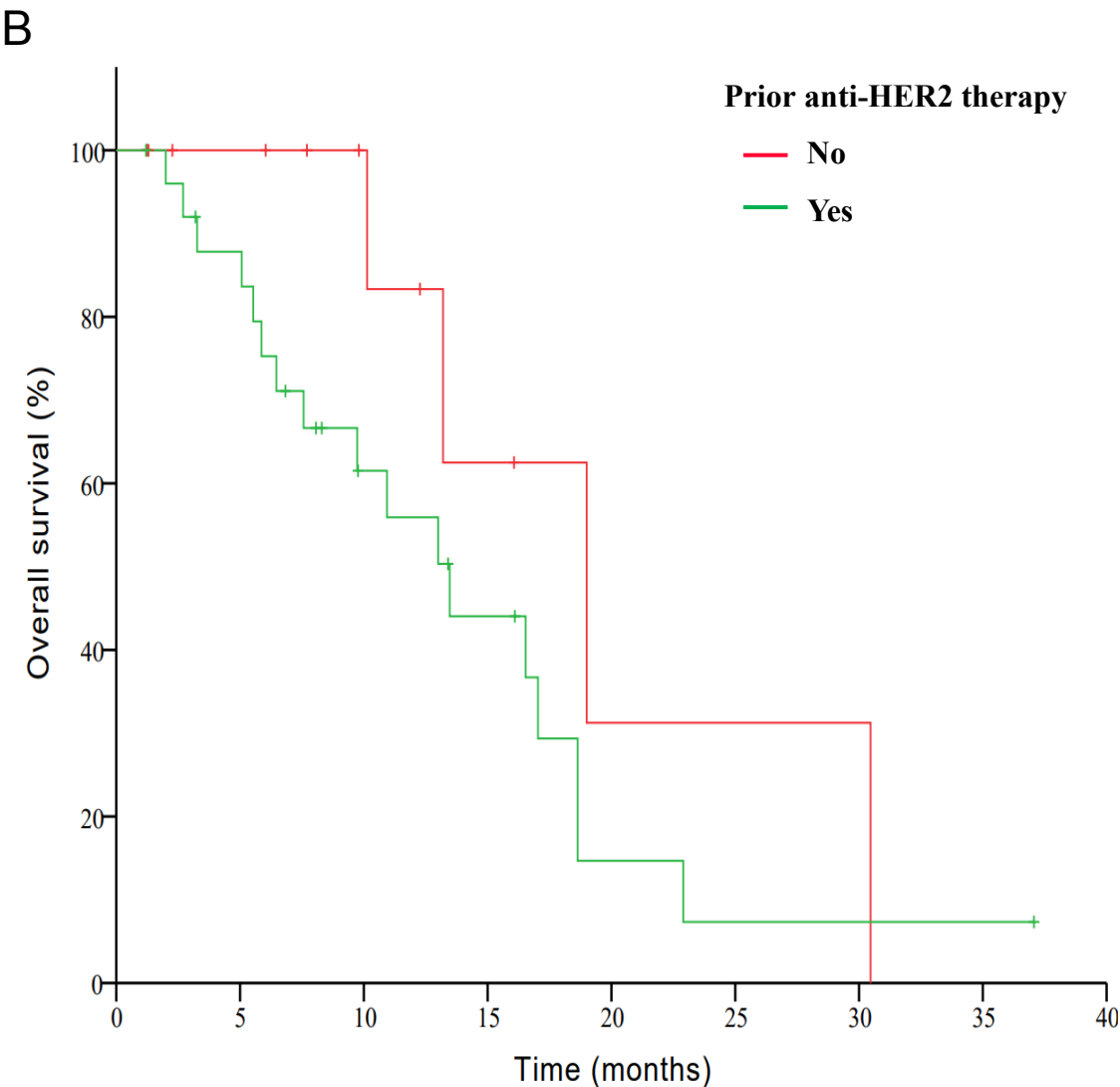

Supplement: Supplementary file 1 [file curroncol-33-00002-s001.zip › curroncol-3992360-supplementary.pdf]
